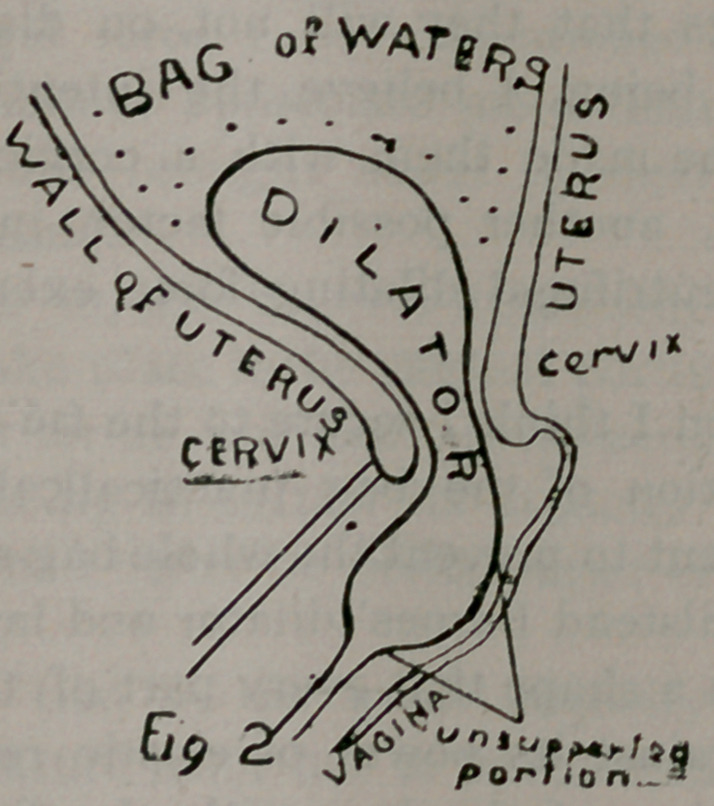# On Some Modifications of Barnes’ Caoutchouc Uterine Dilators

**Published:** 1892-03

**Authors:** Wm. Keiller

**Affiliations:** F. R. C. S. Ed. Univ. Tex. Med. College, Galveston; Professor of Anatomy, University of Texas, Galveston, Texas; late Lecturer on Anatomy, School of Medicine, Edinburgh, and Physician for Diseases of Women to the Edinburgh Providend Dispensary; Fellow of the Obstetrical Society of Edinburgh


DANIEL'S

### TEXAS MEDICAL JOURNAL.

A Representative Organ of the Medical Profession, and an Exponent of Rational Medicine; devoted to the Organization, Advancement and Elevation of the Profession in Texas.

PUBLISHED MONTHLY.—SUBSCRIPTION $2.00 A YEAR.

Vol. VII. AUSTIN, MARCH, 1892. No. 9.

### Original Articles.

CONTRIBUTED EXCLUSIVELY TO THIS JOURNAL.

*The Articles in this Department are accepted and published with the understanding that we are not responsible for, nor do we indorse the views and opinions of the writers by so doing.*

For Daniel's Texas Medical Journal.

### ON SOME MODIFICATIONS OF BARNES' CAOUTCHOUC UTERINE DILATORS.

BY WM. KIELLER, F. R. C. S. ED.,

Professor of Anatomy, University of Texas, Galveston, Texas; late Lecturer on Anatomy, School of Medicine, Edinburgh, and Physician for Diseases of Women to the Edinburgh Providend Dispensary; Fellow of the Obstetrical Society of Edinburgh.

[Read before the Galveston County Medical Club, February 1, 1892.]

MANY, perhaps thirty years ago, my kinsman, Dr. Keiller, of Edinburgh, conceived the idea of utilizing the old India rubber air pessary as a cervical dilator in the induction of premature labor, or in the hastening of the first stage, where that was necessary. In his hands, the air pessary did good work, and his first experiments and subsequent experiences were frequently brought before the Edinburgh Obstetrical Society.

Some time later Dr. Barnes took up the idea, and further elaborated it; devised fiddle-shaped dilators, and used water instead of air, and now Barnes' bags, or what we now know as Barnes' hydrostatic caoutchouc dilators, are known throughout the obstetrical world.

Perhaps the fact that the idea of these dilators first emanated from a Keiller's brain, induced me to think more of the matter, on the rare occasions when I have had to use them, than I would otherwise have done; in any case, I have succeeded in modifying Dr. Barnes' pattern in a manner which received so favorable an acceptance from Edinburgh obstetricians that I venture to bring the matter before this Society and American physicians.

My original paper will be found in the *Edinburgh Medical Journal* for July, 1891.

1. The means provided for the introduction of Dr. Barnes' dilator is an eyelet large enough to admit the point of the index finger or a large staff. It is made so large to avoid the risk of perforation. I have reduced this eyelet till it fits the point of a uterine sound. (*a* Fig. 1.) The bulk of the bag is so lessened that the smallest one may be readily introduced whenever the cervix uteri will admit the finger of the accoucheur; and the point of the eyelet is so strengthened that none but a bungler will run any risk of perforating it. The eyelet is short, and easily rendered aseptic. In Steele's bag, the button at the end adds considerably to the bulk of the bag, and the long tube for the sound, and other crevices, tend to lodge septic matter.

2. The fewer accessories we have to carry in an obstetrical bag, the better, and I have therefore substituted for the stopcock and ground adapter of Barnes' pattern, a stopcock with a bulbous extremity (*b* Fig. 1) which can be attached to a piece of rubber tubing to the ordinary Higginson's, or other form of rubber enema syringe.

3. I have strengthened that part of the bag which, when it is in situ, lies in the vagina. (*c* Fig. 1.)

I noted in my first case that when the dilator is in situ the attempt to fully distend the part of the bag in the cervix and uterus only ended in hyperdistension of the section of the bag lying in the vagina; so that in fact from too zealous a use of the syringe one of my bags shows signs of beginning to give way in the lower end.

A little consideration shows that when in situ the bag consists of three portions (see Fig. 2).

1. A part in the uterus surrounded by the uterine walls and membranes; 2, a part in the cervical canal surrounded by the tissue of the cervix; 3, a part in the vagina, surrounded by the vaginal walls except towards the vaginal outlet, where it is free.

If the bag be introduced and then distended with water, the uterine portion will enlarge, thereby increasing the intra-uterine contents and pressure; and being completely surrounded by the uterus and membranes, this section will be supported pretty equally on all sides.

The cervical portion of the dilator being surrounded by the practically rigid cervix, will not become distended transversely, but the rubber will become more stretched in a longitudinal direction, and the water which this part should contain will be accommodated in the vaginal and uterine portions of the bag.

The vaginal section will be most readily distended, more especially towards the original outlet, where it is entirely unsupported. In the posterior part of the vagina it lies so wedged between the uterus and the rigid posterior pelvic wall that its distension will tend to raise the uterus out of the pelvis; or it may become enlarged laterally and posteriorly, so as to fill up available pelvic space before the fœtal head has descended.

Now the bags act, 1st, as uterine irritants—the irritating elements being the presence of a foreign body in the uterus and vagina, the sudden increase of the intra-uterine pressure, and the separation of the membranes. From this point of view it is especially to be desired that matters be so arranged that the uterine portion of the bag may distend most readily. In Dr. Keiller's original use of the globular air pessary, the whole pessary slipped into the uterus, and in this respect it was perhaps superior to the fiddle-shaped dilators of Dr. Barnes. Dr. Keiller's bags, however, only acted as uterine irritants, supplying also admirably the place of the bag of water where that happened to be ruptured before their introduction. This irritating action is probably the most important element in their *modus operandi*, and I feel doubtful whether, in the present form of Dr. Barnes' dilator, there is not a disadvantage rather than an advantage in so shaping the bags that they will not, on distension, slip inside the uterus, this being, I believe, the intention Dr. Barnes had in view when he made them with a cervical constriction. There is, however, another possible factor in their dilating power, namely, a centrifugal dilating force exerted on the cervical tissues.

This action I think I secure to the full extent of rendering the vaginal portion of the bag practically indistensible beyond a point sufficient to prevent the whole bag slipping into the uterus. If you will distend Barnes' dilator and lay it on the table, it will assume such a shape that every part of the wall will be equally stretched against its power of elastic recoil. Now encircle the cervical portion of the bag with the fingers, and compress it; this portion of the walls will become elongated, the rubber stretched more in the longitudinal than its transverse diameter, and a centrifugal force on the part of the bag will be distinctly felt tending to force apart the compressing fingers.

Compress now the whole uterine portion with the right hand, and the water will be accommodated in the vaginal portion, which will readily yield, while a slightly increased centrifugal pressure will be felt by the fingers encircling the cervical section. Do the same thing with my modified dilators, and as distension of the vaginal portion has been made very difficult, the whole force of the compressing hand will be exerted on the cervical constriction. This is very nearly what occurs when the bags are used as uterine dilators. Between the pains there is a constant centrifugal force exerted on the constricting cervix, and during a pain a marked difference can be noted in using the two forms of dilators.

In the case of Barnes' dilator, the vaginal portion will be felt to swell, especially towards the vaginal outlet, and the bag may be forced out of the uterus; in my modification little alteration takes place on the vaginal portion, but the whole force of the pain is exerted in encreasing the centrifugal dilating pressure on the cervix, while the bag will show no tendency to slip out of its place.

I leave out of account, of course, other ailments in cervical dilation, as the œdematous condition of the cervical tissues (which may possibly be increased by the presence of the extra bulk in the lower part of the uterine cavity), and the thinning out of the lower uterine segment. This last probably remains the same, whether nature be left to herself, or aided as indicated.

The constant centrifugal force I have mentioned is undoubtedly small; but no one should appreciate more than the obstetrician the marvelous results brought about by small forces exerted for a considerable time.

Should the bag, while in, use be over distended, rupture, in Barnes' dilator, would take place in the vaginal portion; in mine, it would occur in the uterine portion. It is therefore doubly necessary, before introducing it, to test its capacity by the syringe, and the fluid used had better be boiled water or boric solution.

I am inclined to think that in the great majority of cases in which induction of premature labor has to be resorted to, the use of the flexible bougie is a superfluity. My practice has been to thoroughly disinfect the vagina, dilate the cervix with the index finger (Hegar's or Goodell's dilator may be used if necessary), and at once introduce the smallest bag. Pains soon supervene; the bags must be changed as required, and labor proceeds rapidly.

There is seldom any hanging on for the completion of the first stage till next day, or the following, as with the bougie.

The dilators have been made for me by Mr. Young, Instrument Maker, and by the Medical Supply Association, both of Edinburgh, Scotland.

## Figures and Tables

**Fig 1 f1:**
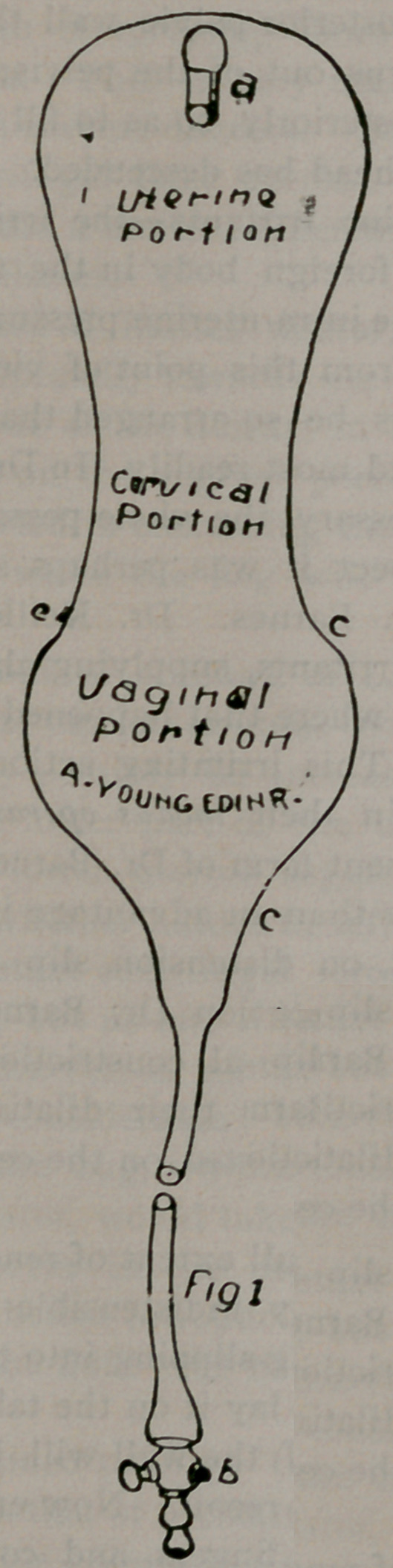


**Fig 2 f2:**